# Optimizing Adjuvant Treatment Recommendations for Older Women with Biologically Favorable Breast Cancer: Short-Course Radiation or Long-Course Endocrine Therapy?

**DOI:** 10.3390/curroncol30010032

**Published:** 2022-12-27

**Authors:** Susan G. R. McDuff, Rachel C. Blitzblau

**Affiliations:** Department of Radiation Oncology, Duke Cancer Center, Durham, NC 27710, USA

**Keywords:** breast cancer, radiotherapy, endocrine therapy

## Abstract

Omission of radiotherapy among older women taking 5 years of adjuvant endocrine therapy following breast conserving surgery for early-stage, hormone sensitive breast cancers is well-studied. However, endocrine therapy toxicities are significant, and many women have difficulty tolerating endocrine therapy, particularly elderly patients with comorbidities. Omission of endocrine therapy among women receiving adjuvant radiation is less well-studied, but available randomized and non-randomized data suggest that this approach may confer equivalent local control and survival for select patients. Herein we review available randomized and non-randomized outcome data for women treated with radiation monotherapy and emphasize the need for future prospective, randomized studies of endocrine therapy omission.

## 1. Introduction

Breast conserving therapy consisting of breast conserving surgery followed by adjuvant radiotherapy (RT) is a well-established alternative to mastectomy for many women diagnosed with early stage breast cancer [1,2]. Adjuvant radiotherapy significantly reduces risk of locoregional recurrence and improves survival from breast cancer [3]. While effective, the daily treatments, traditionally 5–7 weeks in duration, can be inconvenient and are associated with acute and late toxicities. Research efforts focused on improving convenience and reducing radiation toxicity while maintaining efficacy include shorter treatment courses [4,5,6,7,8,9], partial breast radiotherapy [10,11,12,13], and omission of radiotherapy [14,15,16,17] when clinically appropriate. 

Efforts to omit adjuvant radiotherapy have sought to identify a subset of women with favorable, early-stage breast cancer taking endocrine therapy (ET) who may safely avoid adjuvant radiotherapy without compromising oncologic outcomes. Earlier efforts to study radiation omission focused on utilizing clinical and pathologic features, while more modern trials seek to incorporate molecular subtyping and genomic risk stratification to appropriately select patients [18]. However, all of these trials have required patients to take 5 years of adjuvant endocrine therapy. 

Endocrine therapy has proven benefit for women with hormone-sensitive breast cancers [19], and treatment with 5 or more years of adjuvant endocrine therapy has become standard-of-care following breast conserving surgery for those with hormone receptor (HR) positive breast cancers. However, daily endocrine therapy for 5 years can be inconvenient as well, is not without side effects, and many women have significant difficulty tolerating and/or completing endocrine therapy. Recently, there is growing interest in whether a short course of adjuvant radiotherapy may be more tolerable than 5 years of adjuvant endocrine therapy without compromising oncologic outcomes, particularly among older women with low-risk disease. In this review, we summarize existing data studying omission of adjuvant endocrine therapy in favor of utilizing adjuvant radiotherapy alone to treat women with favorable early-stage breast cancer.

## 2. Omission of Radiation Trials

Adjuvant radiotherapy significantly reduces risk of local recurrence and breast cancer specific mortality following breast conserving surgery [3]. However, the absolute risk of recurrence following breast conserving surgery varies with baseline clinicopathologic features and tumor biology, and patients with a higher baseline risk of recurrence following breast conserving surgery benefit more from adjuvant radiation compared to those with a lower risk [3,20,21,22]. The idea that some women with favorable breast cancers taking endocrine therapy after breast conserving surgery may have a sufficiently low risk of recurrence with endocrine therapy alone gave rise to a number of studies investigating omission of radiation in favor of endocrine monotherapy.

CALGB 9343 enrolled 636 women 70 years of age or older between 1994–1999 with cT1N0 estrogen receptor (ER) positive breast cancer treated with breast conserving surgery with negative margins (no tumor on ink) [15,23]. Women were randomized to receive adjuvant tamoxifen with or without adjuvant radiotherapy, prescribed as 45 Gy in 25 fractions to the whole breast and low axilla (levels 1–2), followed by 14 Gy in 8 fractions boost. Tamoxifen was prescribed as 20 mg daily for 5 years. As this study was conducted before sentinel lymph node biopsy became standard, women were allowed to have axillary lymph node dissection (ALND), but this was discouraged and only 36% of the cohort underwent ALND. Women receiving adjuvant radiation had lower rates of locoregional recurrence (breast/low axilla) compared to those receiving adjuvant tamoxifen alone (*p* < 0.001), such that with a median follow up of 12.6 years, 10-year rates of recurrence were 2% in the RT plus tamoxifen arm and 10% in the tamoxifen-alone arm [15]. There were no significant differences in time to mastectomy, time to distant metastasis, breast cancer-specific survival, or overall survival (10-year OS 67% vs. 66%).

PRIME-II enrolled 1326 women aged 65 years or older with low-risk breast cancer between 2003–2009, and were randomized to receive adjuvant endocrine therapy with or without adjuvant radiotherapy [14]. Women were required to have hormone-receptor positive tumors up to 3 cm, pathologically negative axillary lymph nodes, excised with margins 1 mm or greater. Although grade 3 histology and lymphovascular invasion (LVI) (but not both) were permitted, only 3% of women randomized had grade 3 tumors, and 5% had LVI. Patients randomized to radiation received whole breast radiotherapy 40–50 Gy (in 15–25 fractions) and a boost was permitted. The majority received adjuvant tamoxifen, but alternate endocrine therapies were allowed. Updated data presented at the 2020 San Antonio Breast Cancer Symposium showed that 10-year rates of local recurrence were significantly higher among those who did not receive adjuvant radiotherapy (9.8% vs. 0.9%) [24]. Rates of distant metastases (1.4% vs. 3.6%), contralateral breast cancer (1.0% vs. 2.2%), and OS (80.4% vs. 81.0%) were similar between arms [24].

The next generation of radiotherapy omission trials incorporate genomic and molecular subtyping in addition to standard clinicopathologic features to select patients who may be suitable for omission. The LUMINA trial, recently presented at the 2022 American Society of Clinical Oncology meeting, is a Canadian prospective multicenter cohort study that enrolled 501 women 55 years and older between 2013–2017 with luminal A breast cancer to receive 5 years of adjuvant endocrine therapy without radiation following breast conserving surgery [25]. Patients underwent breast conserving surgery with negative margins (≥1 mm) for grade 1–2 tumors up to 2 cm. Luminal A subtype was defined as ER ≥ 1%, PR > 20%, HER2 negative, with Ki67 ≤ 13.25% performed centrally in one of three Canadian laboratories. Median age was 67 and median tumor size was 1.1 cm. At a median follow up of 5 years, 5 year local recurrence was 2.3% (90% CI 1.3, 3.8%) [25]. At this time, it is not clear how recurrence rates will vary as a function of subgroups of interest (e.g., tumor size, patient age, margin status, etc.), and additional follow up is needed. In addition to LUMINA, there are several ongoing trials utilizing molecular and genomic subtyping to select patients for omission of radiation in favor of endocrine monotherapy alone (summarized in Table 1).

Given low absolute rates of recurrence and equivalent survival seen in older women with omission of radiation in CALGB 9343 and PRIME II, current guidelines support omission of radiation for women 70 years and older with ER-positive clinically node negative tumors up to 2 cm who will receive endocrine therapy. However, radiotherapy convenience and toxicity have improved considerably since the publication of these early trials. Hypofractionation is now standard for whole breast radiotherapy [26] and randomized data has matured to support ultra-hypofractionated whole breast regimens [6,27]. Further, many patients with early favorable breast cancers are candidates for partial breast radiotherapy per ASTRO guidelines [28] given the large body of mature randomized data now supporting this approach. Given the toxicity associated with 5 years of endocrine therapy and reduced toxicity of radiotherapy in the modern era, it is important to consider whether a short course of radiotherapy alone may be an acceptable, alternative approach compared to 5 years of endocrine therapy for some older women with early stage, favorable-subtype breast cancer.

## 3. Benefit and Toxicity of Adjuvant Endocrine Therapy

Certainly, endocrine therapy is an important component of treatment for many women with HR-positive breast cancer. The Early Breast Cancer Trialists’ Collaborative Group (EBCTCG) meta-analysis of individual patient data from 20 trials showed that tamoxifen impacts local and distant recurrence, breast cancer specific mortality, contralateral breast cancer, and overall survival. However, the absolute benefit of endocrine therapy depends on absolute breast cancer risk, such that patients with high-risk disease derive more benefit from endocrine therapy compared to those with low-risk disease [19,29,30,31]. Aromatase inhibitors (AIs) are an alternative to tamoxifen for postmenopausal women, and randomized data has shown that treatment with AIs afford reduced recurrence rates and disease free survival compared to tamoxifen [32,33,34]. However, in older women with comorbidities and/or limited life expectancy, benefit of adjuvant endocrine therapy must be weighed against risk of adverse events.

Unfortunately, endocrine therapies are associated with significant short- and long-term side effects such that many women have difficulty tolerating this treatment [35,36]. All forms of endocrine therapy are associated with menopausal symptoms (hot flashes, night sweats, vaginal dryness). Tamoxifen and other selective estrogen receptor modulators (SERMs) additionally can cause venous thrombosis, uterine cancer, cataracts, stroke, vaginal bleeding, nausea, mood perturbance, and weight gain [37]. Letrozole and other aromatase inhibitors can also cause muscle/joint pain, loss of bone density with increased risk for bone fractures, alopecia, cardiovascular disease, hypertension, hypercholesterolemia, and mood perturbance [38,39,40,41]. A recent analysis of 26 studies reveals that adherence to endocrine therapy decreases between the first and fifth year of treatment such that on average only 66% of patients are adherent with therapy by year 5 [42]. Among elderly breast cancer patients with comorbidities, receipt of hormone therapy carries greater risks [43].

Given the acute and late adverse effects of endocrine therapy, it is important to refine methods for selecting patients for either escalation or de-escalation of adjuvant treatment as there may be a subset of women with low-risk early breast cancer for whom the benefit of adjuvant endocrine therapy may not outweigh the risk. Indeed, current European Society of Breast Cancer Specalists/International Society of Geriatric Oncology guidelines for management of older women with breast cancer support omission of endocrine therapy among older women with very low-risk disease and/or short life expectancy in the absence of any documented effect on mortality in this population [44].

A recent analysis of the EORTC 10041/BIG 3-04 MINDACT trial sought to explore outcomes for patients with favorable early-stage HR-positive breast cancer who did not receive adjuvant endocrine therapy [45]. Adjuvant radiotherapy was mandatory for patients who received breast conserving surgery. The MINDACT trial was a phase III trial of 6693 patients with early breast cancer and studied the ability of the MammaPrint 70-gene signature to select women who may benefit from adjuvant chemotherapy [45]. Within this study, a subset of patients were identified as genomically “ultra-low” risk and received no adjuvant systemic therapy. 509 patients with ER-positive, HER2-negative, lymph node negative tumors up to 2 cm who received no adjuvant systemic therapy were matched one-to-one to patients with similar tumor features treated with adjuvant endocrine therapy. At 8 years, distant metastasis free interval (DMFI) rates were 94.8% for women who received no ET versus 97.3% among women treated with adjuvant ET (absolute difference: 2.5%, HR 0.56 (95% CI 0.30–1.03, *p* = 0.057), and overall survival was not different (~95% in both arms). Cumulative incidence of locoregional recurrence and contralateral breast cancer were low in both groups, but slightly higher in those who did not receive ET (4.7% and 4.6%, respectively) compared to those receiving adjuvant ET (1.4% and 1.5%, respectively). These results further support the idea that there may be some women with a sufficiently low risk of distant recurrence that the benefit of adjuvant endocrine therapy might not outweigh the risk.

## 4. Favorable Outcomes Associated with Adjuvant Radiation Monotherapy

While omission of endocrine therapy is less well-studied than omission of radiotherapy, a few randomized studies have demonstrated no difference in overall survival and distant recurrence among select women treated with adjuvant RT monotherapy versus ET monotherapy [46,47,48]. The NSABP B21 trial enrolled 1009 women with node-negative tumors up to 1 cm following breast conserving surgery between 1989 and 1996 to receive RT plus tamoxifen, tamoxifen monotherapy or RT monotherapy after breast conserving surgery [46]. At 8 years, in breast tumor recurrence was highest among women who received tamoxifen alone (16.5%) as compared patients treated with RT alone (9.3%), or combination therapy (2.8%). There was no significant difference in overall survival or distant failures [46].

The British Association of Surgical Oncology (BASO II) trial enrolled 1135 women in the 1990s with low-grade, node-negative tumors up to 2 cm after breast conserving surgery to receive or not receive adjuvant radiotherapy or tamoxifen in a two-by-two factorial design (i.e., RT monotherapy, tamoxifen monotherapy, RT/tamoxifen, or no adjuvant therapy [48]. Both RT (HR 0.37, CI 0.22–0.61 *p* < 0.001) and tamoxifen monotherapy (HR 0.33, CI 0.15–0.70 *p* < 0.004) significantly reduced local recurrence after breast conserving surgery compared to no adjuvant therapy, and the combination of RT and tamoxifen conferred the most reduction in risk [48]. Overall survival at 10 years was 96% with no significant difference between arms [48]. Similarly, the German Breast Cancer Study group randomized 361 patients with low risk, early breast cancer between 1991 and 1998 to receive or not receive adjuvant RT or tamoxifen in a two-by-two factorial design) [47]. Both RT and tamoxifen monotherapy decreased event free survival (EFS) compared to no adjuvant therapy, however in contrast to B21 and BASOII, the combination of tamoxifen with radiation did not improve EFS compared to tamoxifen or radiation monotherapy [47].

Taken together, data from these older randomized studies indicate that there may be a group of women who can safely be treated with adjuvant RT monotherapy, without compromising distant recurrence and survival. In the modern era, researchers at MSKCC retrospectively analyzed outcomes of 888 women 65 years and older with early-stage breast cancer treated with RT alone, ET alone, both or neither following breast conserving surgery between 2010–2015 [49]. 5-year locoregional recurrence was 11% among women receiving no adjuvant therapy, 3% for those treated with ET alone, 4% for RT alone, and 1% for patients receiving both. Compared to patients receiving no adjuvant treatment, RT- and ET-alone afforded similar reduction in locoregional recurrence. Breast cancer specific survival and distant recurrence were not significantly different between groups [49]. Similarly, Joseph et al. reported retrospective outcomes from a Canadian cohort of 1166 patients 70 and older treated between 2005–2015 following breast conserving surgery for low-risk breast cancers with no adjuvant therapy, RT alone, ET alone, or RT and ET [50]. Compared to no adjuvant treatment, RT monotherapy afforded a significant reduction in breast cancer relapse (HR 0.17, *p* < 0.001), similar to ET monotherapy (HR 0.41, *p* = 0.007), and RT and ET (HR 0.24, *p* < 0.001) [50].

In a similar vein, modern population studies likewise reveal favorable outcomes and no difference in overall survival for older women treated with adjuvant RT monotherapy compared to ET monotherapy. A recent analysis of the National Cancer Database (NCDB) included 2995 women 70 and older treated between 2010–2014 following breast conserving surgery for early-stage hormone-receptor-positive, HER2-negative breast cancers treated with adjuvant ET or RT [51]. No difference in overall survival was found among women treated with ET or RT monotherapy [51]. A recent surveillance, epidemiology, and end results (SEER) database analysis investigated outcomes for 13,321 women age 66 and older who underwent breast conserving surgery for early-stage breast cancer between 2007 and 2012 and received either ET and RT, ET alone, RT alone, or no adjuvant therapy [52]. Relative to patients receiving both adjuvant therapies, patients receiving no treatment and ET monotherapy had higher second breast cancer events, whereas RT monotherapy was not associated with an increase in second breast cancer events [52]. Taken together, these studies provide compelling data to support further efforts to study omission of endocrine therapy in favor of adjuvant radiation monotherapy, particularly in older women.

## 5. Ongoing Trials

To this end, the EUROPA trial (Exclusive Endocrine Therapy or Partial Breast Irradiation for Women 70 and Older with Luminal A-like Early-Stage Breast Cancer; NCT04134598) is an ongoing randomized study designed to compare treatment with ET or RT monotherapy in older women with early-stage, favorable breast cancers [53]. Following breast conserving surgery (either with or without sentinel lymph node evaluation), patients with luminal A tumors (defined as ER ≥ 10%, PR > 20%, HER2 negative, with Ki67 < 20%) are randomized to treatment with partial breast radiotherapy alone versus endocrine therapy alone. The primary objectives are to determine patient-reported quality of life (QOL) and demonstrate non-inferior local control. Secondary endpoints will include distant recurrence, adverse events, breast cancer specific survival, and overall survival. The study investigators hypothesize that patients treated with a short course of partial breast radiotherapy will have non-inferior local control, improved quality of life, and be able to avoid the long-term toxicity of adjuvant endocrine therapy [53]. The study plans to accrue 584 women, with an interim analysis planned after 152 patients reach 2 years of follow up [53].

In Canada, the REaCT-70 trial (Randomized, Multicentre Trial Evaluating Harms and Benefits of Endocrine Therapy in Patients ≥ 70 Years of Age with Lower Risk Breast Cancer; NCT04921137) is also investigating feasibility of endocrine therapy omission among patients with low risk breast cancer treated with standard locoregional therapy (breast conserving surgery followed by adjuvant radiotherapy or mastectomy). The study plans to enroll 100 women with axillary node-negative, hormone receptor-positive, HER2- tumors with grade 1 tumors ≤ 5 cm, grade 2 tumors ≤ 3 cm, or grade 3 tumors ≤ 1 cm. Patients will be randomized to receive 5 years of adjuvant endocrine therapy or no endocrine therapy. Outcomes assessed include assessment of adverse events/toxicity and health-related quality of life.

## 6. Conclusions

Adjuvant radiotherapy consistently reduces risk for locoregional recurrence in randomized studies following breast conserving surgery. Given potential toxicity and cost of treatment, research efforts have focused on identifying women who can safely omit radiation. Among women 70 and older with hormone sensitive, early-stage breast cancers, omission of radiation is an accepted treatment approach per NCCN guidelines if adjuvant endocrine therapy is pursued. However, many women have difficulty tolerating endocrine therapy due to toxicity, particularly among older women with comorbidities, resulting in significant drop-out rates. Randomized and non-randomized data summarized here demonstrate favorable rates of local recurrence, distant relapse, and overall survival in this patient population with treated with radiation monotherapy. Given improved convenience and toxicity associated with modern hypofractionated and partial breast radiotherapy approaches, some patients may prefer a radiation monotherapy approach. Ongoing and future prospective trials will allow improved individualization of adjuvant therapy for future patients.

## Figures and Tables

**Table 1:** Select ongoing clinical trials of omission of radiation in favor of endocrine therapy alone for women with early-stage hormone sensitive breast cancers following breast conserving surgery.

Study Title	Eligibility Criteria	Intervention	Primary Outcome	NCT Number	Country	Status	Ph	Est. N
**Randomized**								
DEBRA (De-Escalation of Breast Radiation Trial for Hormone Sensitive, HER2-Negative, Oncotype Recurrence Score Less Than or Equal to 18 Breast Cancer; NRG BR007)	50–70 years, pT1N0, HR+/HER2-, oncotype ≤ 18, treated with lumpectomy and axillary evaluation	Randomized to endocrine therapy with or without adjuvant radiation	IBTR at 5 years	NCT04852887	United States	Recruiting	III	1670
EXPERT (Examining Personalized Radiation Therapy for Low-Risk Early Breast Cancer)	≥50 years, pT1N0, HR+/Her2-, low Prosigna (PAM50) assay, treated with lumpectomy and axillary evaluation	Randomized to endocrine therapy with or without adjuvant radiation	Local recurrence at 10 years	NCT02889874	Australia, New Zealand	Recruiting	III	1167
**Prospective, Non-Randomized**								
PRIMETIME	≥60 years, T1, N0, grade 1–2, HR+/Her2-, central Ki67 testing with very low IHC4+C score treated with lumpectomy and SLNB	Endocrine monotherapy (no adjuvant radiation)	IBTR at 5 years		United Kingdom	Recruiting	III	1500
IDEA (Individualized Decisions for Endocrine Therapy Alone)	50–69 years, pT1N0, HR+/HER2-, oncotype ≤ 18, treated with lumpectomy and axillary evaluation	Endocrine monotherapy (no adjuvant radiation)	Locoregional recurrence at 5 years	NCT02400190	United States	Active, Not Recruiting	II	202
PRECISION (Profiling Early Breast Cancer for Radiotherapy Omission)	50–75 years, pT1N0, HR+/Her2-, grade 1–2 tumors with low Prosigna (PAM50) assay, treated with lumpectomy and axillary evaluation	Endocrine monotherapy (no adjuvant radiation)	Locoregional recurrence at 5 years	NCT02653755	United States	Active, Not Recruiting	II	672

Abbreviations. Ph, phase; N, number; Est., estimated; HR+, hormone receptor positive; ER+, estrogen receptor positive; PR+, progesterone receptor positive; HER2-: human epidermal growth factor receptor 2 negative; IHC4+C score; immunohistochemistry 4 plus clinical score; n/a, not applicable; SLNB, sentinel lymph node biopsy; IBTR, ipsilateral breast tumor recurrence.
